# Artificial 64-Residue HIV-1 Enhancer-Binding Peptide Is a Potent Inhibitor of Viral Replication in HIV-1-Infected Cells

**DOI:** 10.1155/2011/165871

**Published:** 2011-09-19

**Authors:** Mouhssin Oufir, Leslie R. Bisset, Stefan R. K. Hoffmann, Gongda Xue, Stephan Klauser, Bianca Bergamaschi, Alain Gervaix, Jürg Böni, Jörg Schüpbach, Bernd Gutte

**Affiliations:** ^1^Biochemisches Institut, Universität Zürich, Winterthurerstrasse 190, CH-8057 Zürich, Switzerland; ^2^Pharmazentrum Universität Basel, Klingelbergstrasse 50/70, CH-4056 Basel, Switzerland; ^3^Swiss National Center for Retroviruses, Institute for Medical Virology, University of Zürich, Winterthurerstrasse 190, CH-8057 Zürich, Switzerland; ^4^Friedrich Miescher Institute for Biomedical Research, Maulbeerstrasse 66, P.O. Box 2543, CH-4002 Basel, Switzerland; ^5^Département de Pédiatrie, Hôpital des Enfants HUG, CH-1211 Genève, Switzerland

## Abstract

An artificial HIV-1 enhancer-binding peptide was extended by nine consecutive arginine residues at the C-terminus and by the nuclear localization signal of SV40 large T antigen at the N-terminus. The resulting synthetic 64-residue peptide was found to bind to the two enhancers of the HIV-1 long terminal repeat, cross the plasma membrane and the nuclear envelope of human cells, and suppress the HIV-1 enhancer-controlled expression of a green fluorescent protein reporter gene. Moreover, HIV-1 replication is inhibited by this peptide in HIV-1-infected CEM-GFP cells as revealed by HIV-1 p24 ELISA and real-time RT-PCR of HIV-1 RNA. Rapid uptake of this intracellular stable and inhibitory peptide into the cells implies that this peptide may have the potential to attenuate HIV-1 replication *in vivo*.

## 1. Introduction

Treatment of HIV-1 infection has made considerable progress. Nevertheless, all current therapeutic approaches have been hampered by viral resistance mutations, undesired side effects, and high treatment cost [1].

Therefore, additional concepts for inhibiting HIV-1 replication, particle assembly, and escape have been developed.

One approach to interfere with HIV-1 infection at an early stage is to block viral entry. Enfuvirtide, a synthetic peptide already in clinical use corresponding to the conserved extramembranous sequence 643 to 678 of the HIV-1 envelope glycoprotein gp41, was found to prevent the conformational change of gp41 necessary for HIV-1 fusion with CD4^+^ cells [2, 3]. Other approaches include the construction of a highly colonizing *Escherichia coli* (*E. coli)* strain secreting HIV-1 gp41-hemolysin to block HIV fusion with target cells [4], the use of an interchain disulfide bond-stabilized trimeric 45-residue fusion peptide [5, 6] to inhibit the fusion of a large number of HIV isolates with target cells, and the inhibition of HIV-1 infection of CD4^+^ T cells by microbial HSP70 [7].

Similarly, the human monoclonal 5H/I1-BMV-D5 single-chain variable region fragment (scFv) antibody selected from phage-displayed libraries has been shown to bind to the N-terminal heptad repeat region of gp41 and thus inhibited the formation of fusion intermediates *in vitro* and the replication of diverse clinical isolates of HIV-1 [8]. In macaques, protection from vaginal challenge with simian human immunodeficiency virus was achieved by vaginally delivered inhibitors of virus-cell fusion; moreover, two small molecule inhibitors binding to viral gp120 and cellular CCR5, respectively, prevent receptor binding by gp120, and a 53-residue peptide containing the C-terminal heptad repeat region of gp41 inhibited the gp41-mediated virus-cell fusion [9].

However, HIV-1 particles interacting only weakly with fusion inhibitors would still be able to enter target cells [10]. This requires other strategies to repress the viral replication after the virus has invaded the target cells. These include Highly Active AntiRetroviral Therapy (HAART) treatment [1], the intracellular expression of an antisense gene targeting the HIV-1 envelope [11] and the sequence-specific targeting of the three Sp1 sites of the 5′-long terminal repeat (LTR) of HIV-1 by engineered zinc-fingers [12]. In the present work, we have designed a 64-residue protein that crossed plasma and nuclear membranes, recognized the two highly conserved NF-*κ*B binding sites (enhancers) of the 5′-LTR of HIV-1, and acted as a strong inhibitor of HIV-1 replication in infected cells.

**Figure 1:**
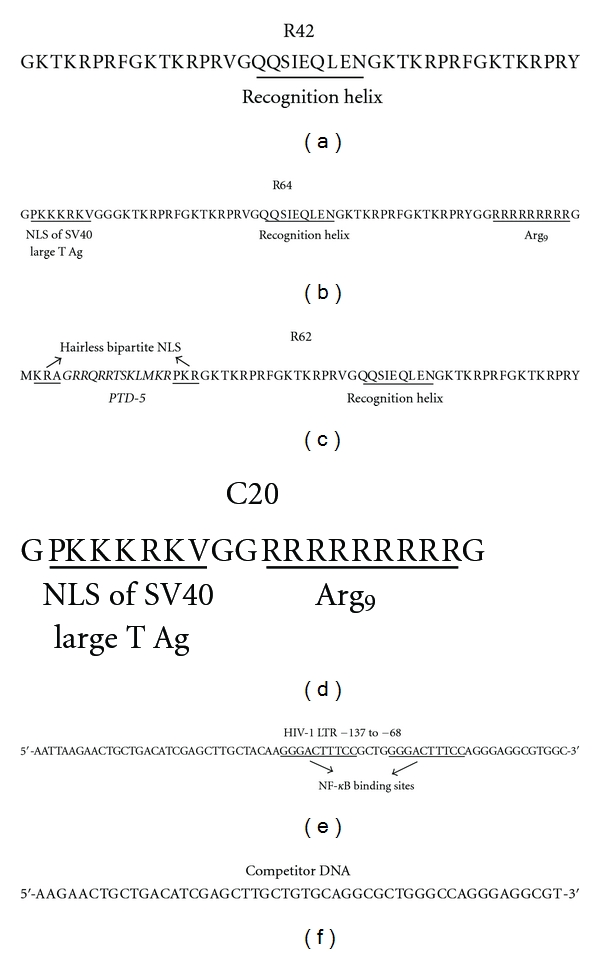
Relevant peptide and DNA sequences. (a) 42-residue HIV-1 enhancer-binding peptide (R42) derived from the DNA-binding domain of 434 repressor. The recognition helix in (a), (b), and (c) is underlined. (b) 64-residue HIV-1 enhancer-binding peptide obtained through extension of R42 by the nuclear localization signal of SV40 large T antigen and nona-arginine (NLS and Arg_9_ underlined). (c) 62-residue HIV-1 enhancer-binding peptide obtained through extension of R42 at the N-terminus by the bipartite nuclear localization signal of Hairless (underlined) embracing protein transduction domain-5 (PTD-5, italics). (d) 20-residue control peptide containing only NLS of SV40 large T antigen and Arg_9_ linked by two glycine residues (NLS and Arg_9_ underlined). (e) 70-bp HIV-1 LTR DNA comprising positions −137 to −68. The two identical NF-*κ*B-binding sequences (enhancers) are underlined. (f) 51-bp competitor DNA lacking the two enhancers, otherwise it is largely identical with the 70-bp HIV-1 LTR DNA shown in (e).

This synthetic 64-residue peptide (R64, Figure 1) contained, from N- to C- terminus, the nuclear localization signal of SV40 large T antigen, the recognition helix of bacteriophage 434 repressor (residues 28 to 36) flanked at both ends by two copies of the positively charged repressor sequence 37 to 44 and nine arginine residues (Arg_9_) for plasma membrane transduction [13]. The recognition helix of 434 repressor was used because the nucleotide sequences of the two enhancers in the 5′-LTR region of HIV-1 were identical to repressor-binding sequences of the 434 operators, and shorter versions of R64 lacking Arg_9_ [14] or both the Arg_9_ and the NLS sequences (R42, [14, 15]) had been shown to bind to the enhancer region of synthetic HIV-1 LTR DNA, thereby displacing the p50 subunit of NF-*κ*B from its two binding sites in LTR and inhibiting HIV-1 enhancer-driven transcription [14, 15].

Despite their potential immunogenicity, therapeutic peptides may play an important role as shown by their application in the treatment of prostate cancer in mice [16], in the prevention of HIV-1 infection [2, 3], and the induction of Wilms' tumor-gene-(WT1)-specific cytotoxic T lymphocytes with resultant cancer regression in human [17]. Since covalently linked oligoarginine and related sequences efficiently mediate membrane transduction of polypeptides [18, 19], R64 and similar peptides could be very useful in a combinatorial therapy against HIV-1 infection by targeting the NF-*κ*B-binding sites of the HIV-1 enhancers [20].

## 2. Materials and Methods

### 2.1. Synthesis and Purification of the Peptides

The 20 to 64 residues long peptides were synthesized by the solid-phase method [22, 23] on an Applied Biosystems 430A peptide synthesizer using Fmoc chemistry and following the instructions of the manufacturer. After deprotection and cleavage from the resin, they were purified on an Agilent 1100 HPLC system equipped with an autosampler, a diode array detector and an Interchim Uptisphere C18 column (250 × 4.6 mm). The peptides were eluted with a solvent gradient from 0.1% trifluoroacetic acid (TFA) to 0.075% TFA in acetonitrile. The purity of the peptides was demonstrated by ion spray mass spectrometry on a MALDI-TOF Bruker spectrometer and their concentration determined by quantitative amino acid analysis on a Biochrom 20 amino acid analyzer.

All synthetic oligodeoxyribonucleotides were purchased from MicroSynth (Balgach, Switzerland).

### 2.2. Band Shift Assays of R64-HIV LTR Interactions

**Figure 2:**
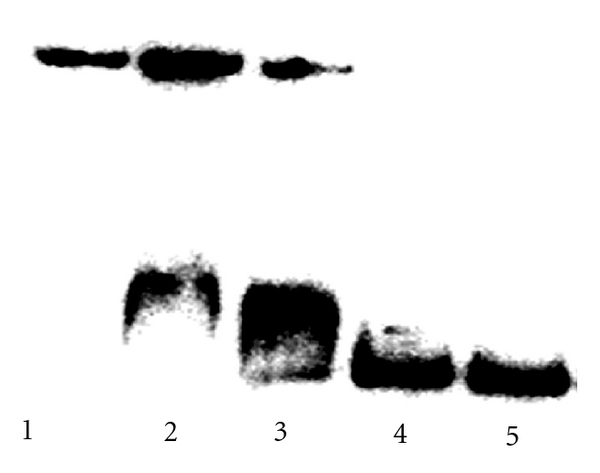
Competitive band shift assays of the interactions of R64 with ^32^P-labeled HIV-1 LTR DNA (Figure 1(e)) in the presence of increasing amounts of unlabeled competitor DNA (Figure 1(f)). Samples (20 *μ*L each) were incubated in 50 mM phosphate buffer, pH 7.4, for 30 min at 37°C [21] and then submitted to polyacrylamide gel electrophoresis at 250 V in 33 mM Tris, 0.75 mM EDTA, adjusted to pH 8.2 using boric acid. R64; lanes 1 to 4 (8 pmol each); competitor DNA; lanes 1 to 4 (1, 4.25, 8.5, 17 pmol, resp.); labeled enhancer-containing DNA (target DNA); lanes 1 to 5 (200 fmol each).

Assays followed standard procedures [21, 25]. Binding reactions, performed in triplicate with identical results, contained R64 (Figure 1(b)), a synthetic 5′-end ^32^P-labeled 70-base pair (bp) oligodeoxyribonucleotide (Figure 1(e)) enclosing the two NF-*κ*B-binding sequences (enhancers, underlined residues), and a synthetic 51-bp competitor DNA (Figure 1(f)) in 20 *μ*L phosphate buffer, pH 7.4 [26]. After incubation for 30 min at 37°C, samples were analyzed by 10% polyacrylamide gel electrophoresis. After drying, the radioactivity in the gel was analyzed by phosphoimaging (Figure 2).

### 2.3. Footprint Assay of R64-HIV-1 Enhancer-Binding Specificity

**Figure 3:**
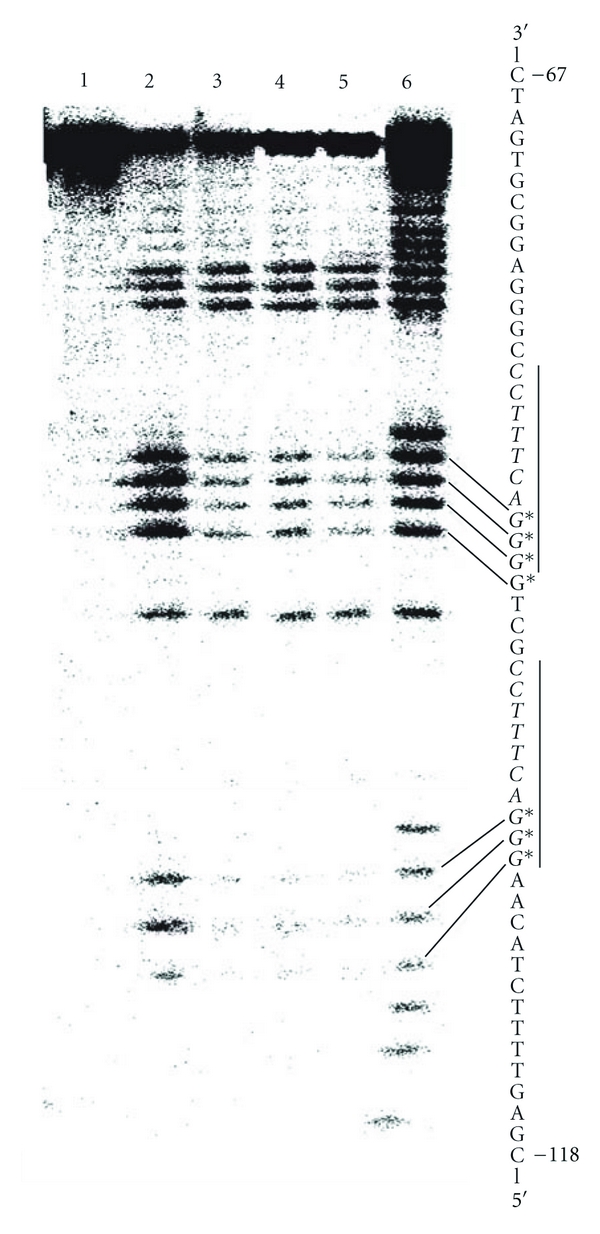
Footprint assay of the specificity of R64-HIV-1 enhancer binding. The two enhancer sequences are marked by vertical bars. Lanes 1 to 5 contained 1 pmol each of labeled HIV-1 LTR DNA (positions –118 to –67), which had been methylated by dimethyl sulfate and cleaved by piperidine in lanes 2 to 5. Lanes 3 to 5 contained R64 (50, 100 and 200 pmol, resp.) and competitor DNA (200 pmol per lane). Lane 6, G/A bands of Maxam-Gilbert sequencing [24] of the HIV-1 LTR DNA. G*, hyporeactive G bases.

The synthetic HIV LTR DNA (sequence shown in Figure 3) comprised positions –118 to –67 including two enhancer regions (Figure 3, vertical bars). The standard footprint procedure [27] was modified as follows. R64 was added to the labelled truncated HIV-1 LTR DNA and the mixture submitted to gel electrophoresis. The bands of the R64-DNA complex and of free DNA were excised, and sterically available N-7 positions of the guanine bases were methylated in the gel slices using dimethyl-sulfate [28]. After elution from the gel slices the DNA was cleaved at the sites of methylation by 10% piperidine [24], and the fragments formed were resolved by gel electrophoresis in a buffer containing 90 mM Tris-borate, pH 8.3, 25 mM EDTA. Dried gels were analyzed by phospho-imaging (Figure 3). In parallel, sequencing of the labeled DNA by the method of Maxam and Gilbert [24] allowed localization of the binding sites of R64. The footprint assay was performed twice; the patterns obtained were in very good agreement.

### 2.4. HIV-1 LTR-Controlled *In Vitro* Transcription in the Absence and Presence of R64

**Figure 4:**
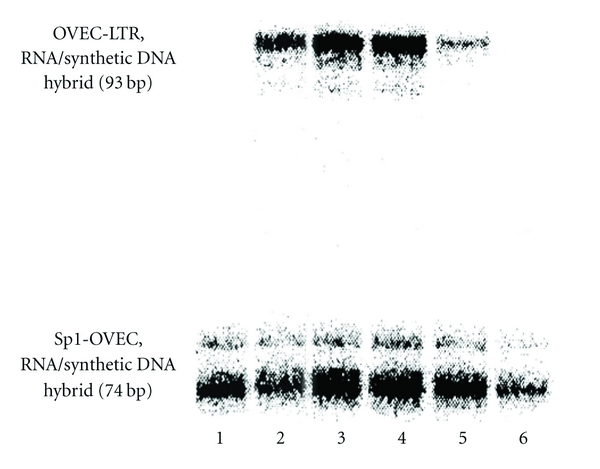
Gel electrophoretic analysis of the effect of R64 on *in vitro* transcription of OVEC-LTR and Sp1-OVEC in HeLa cell nuclear extract (OVEC-LTR: target plasmid containing the two enhancer sequences of HIV-1 LTR and a *β*-globin reporter gene; Sp1-OVEC: control plasmid lacking the enhancer sequences and the first 19 bp of the *β*-globin reporter gene). Transcription experiments containing the two plasmids, 100 ng each, and increasing amounts of R64 were performed as described in [14]. The total volume per experiment was 25 *μ*L. Transcripts were hybridized with a labeled synthetic 93-mer DNA, corresponding to positions –18 to +75 of the *β*-globin reporter gene and then treated with nuclease S1. The resulting digests were submitted to gel electrophoresis in Tris-borate (89 mM, pH 7.5), EDTA (2 mM), urea (7 M). The gel shows the undigested RNA/DNA hybrids obtained. Lane 1: Sp1-OVEC; lanes 2 to 6: OVEC-LTR + Sp1-OVEC in the presence of 0, 16, 32, 65 and 97.5 pmol of R64, respectively.

OVEC-LTR, the plasmid used to test the activity of R64 in cell-free *in vitro* transcription, contained the two NF-*κ*B and the three specificity protein 1 (Sp1) binding sites (positions –110 to –45) of HIV-1 LTR and a *β*-globin reporter gene [14, 29]. As a control, plasmid Sp1-OVEC contained only Sp1 binding sites and lacked the first 19 base pairs of the *β*-globin gene [29]. Equal amounts of both plasmids were used (100 ng each) and the protocol described previously was followed [14]. RNA extraction, purification [30], and nuclease S1 digestion in the presence of a labeled 93-residue oligodeoxyribonucleotide [29, 31] (corresponding to positions −18 to +75 of the coding strand of the *β*-globin reporter gene of OVEC-LTR) were followed by denaturing gel electrophoresis of the digests and phospho-imaging of the gel (Figure 4). The *in vitro* transcription experiments were performed in triplicate with nearly identical results.

### 2.5. Analysis of the Intracellular Localization of R64

**Figure 5:**
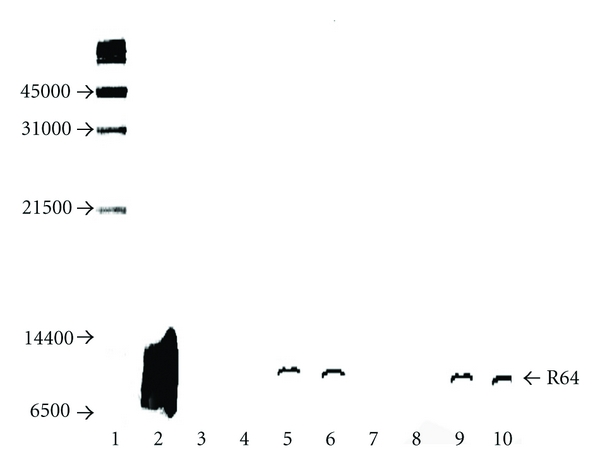
Western blot of cytosolic and nuclear extracts of R64-containing CHO-K1 cells. R64 was detected using rabbit anti-R42 antiserum, alkaline phosphatase-conjugated goat anti-rabbit IgG, and the phosphatase substrate 5-bromo-4-chloro-3-indolyl phosphate. Lane 1: molecular mass markers; lane 2: synthetic R64 (calculated molecular mass, 7611 Da; 500 ng applied); lanes 3 and 7: nuclear and cytosolic cell extract controls; nuclear and cytosolic extracts of cells incubated with 1.3 *μ*M R64 (lanes 4 and 8), 1.7 *μ*M R64 (lanes 5 and 9), and 2.3 *μ*M R64 (lanes 6 and 10), respectively.

CHO-K1 cells (~10^5^ cells) containing different concentrations of R64 were incubated at 37°C for 24 h. The cells were then harvested and treated with low-salt lysis buffer (containing 20 mM KCl) at 4°C for 5 minutes. The cytosolic extract was obtained by centrifugation. The pellet was incubated with high-salt lysis buffer (containing 1.2 M KCl) at 4°C for 5 minutes and sonicated for 2 minutes to give the nuclear extract. Both extracts were analyzed by standard electrophoresis [32] and Western blotting [33] and R64 was detected colorimetrically after addition of polyclonal rabbit anti-R42 antibodies, incubation with goat anti-rabbit IgG-conjugated alkaline phosphatase, and addition of nitrotetrazolium blue chloride and the p-toluidine salt of the 5-bromo-4-chloro-3-indolyl phosphate substrate (Sigma) [13, 34] (Figure 5). The experiment to study the intracellular localization of R64 was performed twice with almost identical results. 

### 2.6. Incubation of R64 (Figure 1(b)) and R42 (Figure 1(a)) with Phorbol-12-Myristate-13-Acetate (PMA)-Stimulated CEM-GFP Cells

**Figure 6:**
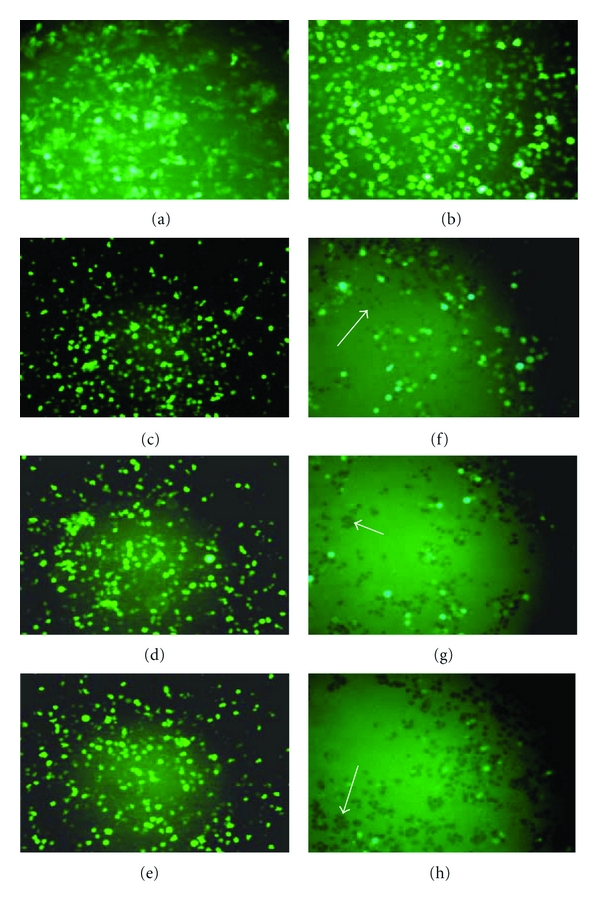
Fluorescence microscopy of CEM-GFP cells. (a) unstimulated cells; (b) CEM-GFP cells stimulated with 0.32 *μ*M PMA for 16 h at 37°C; (c, d, e) CEM-GFP cells stimulated with 0.32 *μ*M PMA in the presence of 4 *μ*M, 8 *μ*M, and 12 *μ*M R42, respectively, for 6 h at 37°C; (f, g, h) treatment of CEM-GFP cells with 0.32 *μ*M PMA in the presence of 2.6 *μ*M, 5.25 *μ*M and 7.9 *μ*M R64, respectively, for 6 h at 37°C drastically reduced the number of cells expressing the GFP gene; the arrows point to representative cells in which the GFP gene was silenced.

CEM-GFP cells [35] contain a plasmid encoding a green fluorescent protein (GFP) reporter gene under HIV-1 LTR control. Both HIV-1 and PMA stimulate HIV-1 enhancer-linked transcription via activation of NF-*κ*B resulting in high level expression of GFP [36, 37]. CEM-GFP cells were maintained in 2 mL RPMI-1640 medium supplemented with 100 U/mL penicillin, 100 *μ*g/ml streptomycin, 200 mM L-glutamine, 24 mM sodium bicarbonate, and 10% heat-inactivated fetal calf serum (Life Technologies) in a 24-well culture plate for 24 h. Then PMA (200 ng per mL) and increasing amounts of peptides R64 or R42 were added per well and the change of fluorescence was observed in a Nikon Diaphot 300 fluorescence microscope using a 485 nm excitation filter and a 535 nm emission filter (Figure 6).

After 48 h, cell growth-inhibitory effects of the peptides and PMA were assessed using the 3-[4,5-dimethylthiazol-2-yl]-2,5-diphenyltetrazolium bromide (MTT) assay [38].

### 2.7. Incubation of Peptides R64, R62, R42, and C20 with HIV-1-Infected CEM-GFP Cells

R62 differed from R64 only in the arrangement of the membrane transduction signals; R62 (Figure 1(c)) contained both the nuclear localization signal (NLS) and the plasma membrane protein transduction domain (PTD) at the N-terminus of the peptide chain. C20 (Figure 1(d)), a 20-residue control peptide, comprised the NLS and PTD sequences of R64, linked by two glycine residues.

HIV-1 stocks were prepared from cultures of human H9 lymphoblastoid cells (NIH AIDS Research and Reference Reagent Program) maintained in complete medium (RPMI-1640 supplemented with 2 mM L-glutamine, 24 mM sodium bicarbonate, and 10% heat-inactivated fetal calf serum (Life Technologies)) and chronically infected with HIV-1 strain III B (HIV-1_IIIB_). HIV-1-containing supernatant was filtered through a 0.2 *μ*m filter, aliquoted, and stored at −70°C until used.

Human CEM-GFP cells were in an exponential growth phase before being pelleted in 50 mL conical tubes (Falcon) to give ~9.6 × 10^6^ cells per tube.

Stocks of HIV-1_IIIB_ obtained from the supernatant of human H9 T cells were diluted 1 : 5 with complete medium, a concentration previously determined to give a continuous rate of HIV replication (data not shown). The CEM-GFP cell pellets were incubated with 1-ml aliquots of diluted HIV-1 and gently resuspended. After incubation at 37°C for 2 h, excess virus was removed and the cells were thoroughly washed with RPMI-1640 medium.

Cells were suspended in 48 mL of complete medium at a concentration of 2 × 10^5^ cells per mL, and 2-ml aliquots of either HIV-1-infected or mock-infected cells were transferred to a 24-well Falcon tissue culture plate thus containing 4 × 10^5^ cells per well. The peptides R64, R62, R42, and C20 were added in duplicate to the wells at final concentrations of 0, 0.1, 0.25, 0.5, 1.0, 1.5, 2.0, 2.5, 3.0, 3.5, and 7 *μ*M, respectively.

At different time points, the HIV-1 p24 core protein in the cell-free culture supernatants of the wells was determined by quantitative ELISA as described previously [10]. Briefly, duplicate supernatants (25 *μ*L each) of the cultures of the HIV-1-infected, peptide-treated CEM-GFP cells were diluted 1 : 6 with buffer and then added to microplate wells precoated with a mouse monoclonal anti-p24 antibody. This was followed by addition of a biotinylated human polyclonal anti-p24 antibody and incubation with streptavidin-horseradish peroxidase conjugate. Additional binding sites for streptavidin-horseradish peroxidase, created by treating the wells with biotinyl-tyramide [40], were reacted with a dilute solution of streptavidin-horseradish peroxidase as recommended by the manufacturer. Finally, the o-phenylene diamine substrate was added and the resulting yellow color measured spectrophotometrically at 490 nm. p24 was quantified by comparing the absorbances of the samples with those of a standard curve obtained from p24 concentrations between 0.17 pg/mL and 10 ng/mL.

To determine the HIV-1 RNA levels in the culture supernatants, quantitative real-time RT-PCRs specific for the gag region of HIV-1 were performed as described in [41] using 1 *μ*L of cell-free culture supernatant derived from the HIV-1-infected, peptide-treated CEM-GFP cells, or 1 *μ*L of an RNA standard dilution series obtained from the supernatant of HIV-1_IIIB_-infected human H9 lymphoblastoid cells and calibrated using the Amplicor (Roche) quantitative RT-PCR assay.

After eight days of incubation of the HIV-1-infected cells with peptides, possible peptide-derived cytotoxic effects were assessed using the MTT assay [38] with the following modifications. The infected CEM-GFP cells grown in the presence or absence of peptide were resuspended, duplicate 100 *μ*L aliquots transferred to a 96-well ELISA plate (Nunc), and 10 *μ*L of a solution of 5 mg MTT per mL PBS added to each well. After a four-hour incubation, 100-*μ*L solubilization solution (10% SDS, 0.5% Triton X-100, 0.01 M HCl) was added per well and the plate further incubated at 37°C overnight. Finally the absorption was measured in an ELISA plate reader (TecScan) at 570 nm with background subtraction at 630 nm.

## 3. Results and Discussion

We have shown earlier that a 42-residue peptide derived from the DNA-binding domain of bacteriophage 434 repressor bound specifically to the enhancer region of HIV-1 long terminal repeat and repressed the *in vitro* transcription of HIV-1 enhancer-containing plasmids [14]. In the present work we have demonstrated that this peptide was active in HIV-1-infected cells after it had been extended by a protein transduction domain to cross the plasma membrane and by a nuclear localization signal to enter the nucleus. The best results were obtained with peptide R64 (Figure 1(b)).

### 3.1. DNA Binding Specificity of R64 and R62 at the Two HIV-1 LTR NF-*κ*B Binding Sites

The binding specificity of R64 and R62 was determined by competitive band shift and footprint assays. Band shift assays in the presence of R64 (Figure 2) showed that, in the experimental conditions chosen, an approximately 100-fold molar excess of competitor DNA (Figure 1(f)) was required to displace the peptide from the enhancer-containing HIV-1 LTR target DNA (Figure 1(e)).

The result of the band shift assay performed in the presence of R62 was very similar (data not shown).

Footprint analysis followed a procedure [42] in which protein-DNA complexes and free DNA were first separated by band shift electrophoresis and then treated with a DNA-cleaving reagent in the gel matrix. The resulting DNA fragments were eluted from the gel and resolved by gel electrophoresis. In the present work, the electrophoretically separated bands of an R64-HIV-1 LTR DNA complex and free HIV-1 LTR DNA were excised from the gel and the DNA in the bands was treated with dimethyl sulfate, eluted from the gel slices, and then incubated with piperidine [24, 28]. The DNA fragments obtained were separated by electrophoresis on a sequencing gel (Figure 3). The results showed that the G bases of the two NF-*κ*B binding sites (enhancers, marked by vertical bars) of HIV-1 LTR were largely protected from methylation in the presence of R64 (Figure 3, lanes 3 to 5) indicating that R64, like R42 [26], could compete with NF-*κ*B for HIV-1 enhancer binding and thus inhibit enhancer-controlled transcription. This was confirmed by the following experiments.

### 3.2. *In Vitro* Transcription Suppression

In HeLa cell nuclear extracts, R64 suppressed the transcription of a reporter gene from an HIV-1 enhancer-containing plasmid (OVEC-LTR) more strongly than the transcription of this gene from a control plasmid lacking the HIV-1 enhancer region (Sp1-OVEC) [14, 29] (Figure 4, lanes 5 and 6). R62, largely identical with R64 except that an N-terminal bipartite nuclear localization signal [43] embraces the protein transduction domain (Figure 1(c)), was less specific in the suppression of HIV-1 enhancer-controlled transcription than R64 despite its lower net positive charge (+25 versus +28, data not shown).

### 3.3. Analysis of the Intracellular Localization of R64

R64 was shown to be present in both the cytosolic and nuclear extract of R64-incubated CHO-K1 cells using a double antibody assay following electrophoresis and Western blotting of the two extracts (Figure 5). R64 was detected when its concentration in the incubation mixture (~10^5^ cells/3 mL medium) was above 1.3 *μ*M. The minute increase in the amount of R64 in the nuclear extracts going from 1.7 *μ*M to 2.3 *μ*M R64 in the incubation mixtures (Figure 5, lanes 5 and 6) indicated that the “receptor” for nuclear protein import [44] was almost saturated at 1.7 *μ*M R64 in the incubation mixture and that excess intracellular R64 remained in the cytosol (Figure 5, lanes 9 and 10).

### 3.4. R64 Suppressed PMA-Stimulated HIV-1 Enhancer-Controlled Transcription

After R64 was shown to be present in the nuclear extract of R64-incubated CHO-K1 cells (Figure 5), it was tested whether this peptide was active in intact CEM-GFP cells [35] which contain an HIV-1 LTR-controlled GFP reporter gene whose expression is stimulated, via activation of NF-*κ*B, by HIV-1, TNF-*α*, interleukin-1, lipopolysaccharide, and PMA [36, 37, 45].

CEM-GFP cells (approximately 10^5^ cells in two mL medium per well) were incubated with PMA (200 ng), which mimicked HIV-1 infection, and increasing amounts (20 to 60 *μ*g) of R42 and R64, respectively. Fluorescence microscopy showed that, in the experimental conditions applied, PMA stimulated GFP gene expression strongly (Figures 6(a) and 6(b)). Addition of R42 lacking the plasma membrane transduction and the nuclear localization signals of R64 had very little if any effect on the fluorescence of the PMA-stimulated CEM-GFP cells up to a concentration of 6 *μ*M (Figures 6(c), 6(d) and 6(e)). R64, however, extinguished the fluorescence of these cells almost completely at a concentration of approximately 4 *μ*M (Figures 6(f), 6(g) and 6(h)) because it was able to enter the nucleus and to inhibit the HIV-1 enhancer-controlled expression of the GFP reporter gene. 

Cytotoxicity in the presence of PMA and increasing concentrations of R42 and R64 was not observed using the MTT assay [38].

### 3.5. R64 Inhibited HIV-1 Replication in CEM-GFP Cells

R64 inhibited the HIV-1 replication in infected CEM-GFP cells in a concentration-dependent manner as shown by quantitative assays of HIV-1 p24 and HIV-1 gag RNA.

**Figure 7:**
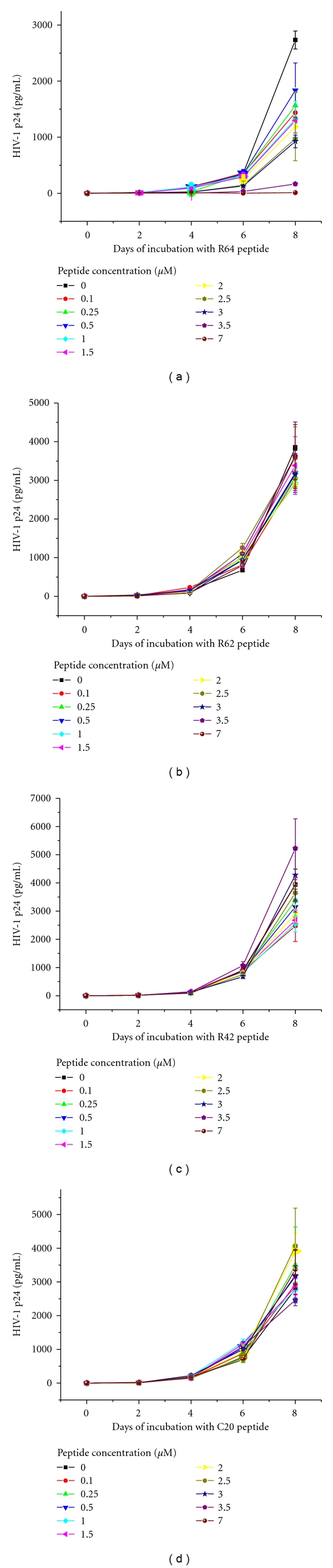
Assessment of HIV-1 p24 levels in the culture supernatants of HIV-infected CEM-GFP cells during eight days of incubation with increasing concentrations of peptides R64 (a), R62 (b), R42 (c), and C20 (d) starting at day one post-infection. The figure shows the results of two independent *in vitro* infection experiments with mean value ± standard deviation.

The HIV-1 p24 ELISA was performed as described [10]. In Figure 7, the results obtained with R64, R62 (Figure 1(c)), R42 (Figure 1(a)), and C20 (Figure 1(d), comprising only the nuclear and the plasma membrane transduction signals of R64) are compared. Noticeable p24 production in the presence of 0–7 *μ*M R64 (Figure 7(a)) started approximately six days after addition of the peptide except in the samples containing 3.5 and 7 *μ*M R64, respectively, in which HIV-1 p24 levels remained close to zero. The production of p24 in the presence of 0–7 *μ*M R62, R42, and C20 (Figures 7(b), 7(c) and 7(d)) started uniformly after approximately five days of incubation and then rose logarithmically without efficient inhibition by these peptides.

Real-time RT-PCR experiments [41] showed that the number of HIV-1 RNA copies per mL supernatant of infected cells dropped by nearly three orders of magnitude in the presence of 7 *μ*M R64 (Figure 9(a)) but remained constantly high in the presence of 7 *μ*M R62, R42 or C20 in the infection experiments (Figures 9(b), 9(c) and 9(d)). At maximal concentration of R64, HIV-1 replication was inhibited more than 300-fold.

Figure 9 also indicates the degree of growth inhibition of the HIV-1-infected CEM-GFP cells in the presence of R64, R62, R42 and C20, respectively. The MTT assay used for this purpose measures metabolically active, viable cells. The maximum of 20% inhibition observed in the presence of R64 (Figure 9(a)) indicates that 80% of the cells are still metabolically active and viable. A loss of only 20% of the metabolic capacity of these cells cannot account for more than 300-fold inhibition of HIV-1 replication observed with R64. 

R62, which differed from R64 only in sequence and arrangement of the nuclear localization and plasma membrane transduction signals, had no antiviral activity in HIV-1-infected CEM-GFP cells. From earlier work we knew that R42 was not able to pass the plasma membrane and therefore was inactive in these assays. C20 presumably reached the nucleus but its sequence did not have enhancer-binding specificity.

**Figure 8:**
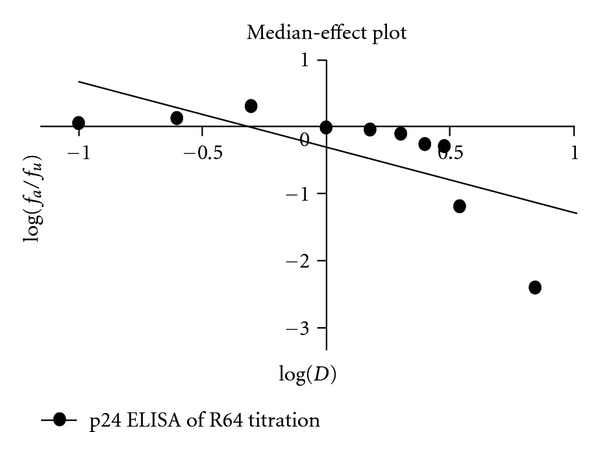
Determination of the 50% effective dose of peptide R64 based on R64 concentration-dependent HIV-1 p24 levels. Using the CalcuSyn 1.1 software package (Biosoft) [39], a median-effect plot was derived from the dose-effect curve obtained at day 8 after infection using the equation: *f*_*a*_/*f*_*u*_ = (*D*/*D*_*m*_)^*m*^ where *D* is the dose, *D*_*m*_ is the dose required for a 50% effect, *f*_*a*_ is the fraction affected by the dose, *f*_*u*_ is the unaffected fraction, *m* is a coefficient of the sigmoidicity of the dose-effect curve, and *r* is the correlation coefficient (*D*_*m*_ = 0.493 *μ*M, *r* = 0.6849, *m* = −0.981).

In Figures 8 and 10, by using the CalcuSyn 1.1 (Biosoft) software package [39], median-effect plots were derived from dose-effect curves obtained for R64 as antiretroviral compound using the median-effect equation:

(1) $$\frac{f_{a}}{f_{u}}={\left(D/D_{m}\right)}^{m},$$

where *D* is the dose, *D*_*m*_ is the dose required for a 50% effect, *f*_*a*_ is the fraction affected by the dose, *f*_*u*_ is the unaffected fraction and *m* is a coefficient of the sigmoidicity of the dose-effect curve. The median-effect dose (*D*_*m*_) was used to describe the 50% effective dose (ED_50_) and in this case, this ED_50_ corresponds to IC_50_ (50% inhibitory concentration) which is the drug level needed to block 50% of HIV's normal replication *in vitro.*

R64 peptide titrations of HIV-1-infected CEM-GFP cells analyzed by p24 ELISA and one tube real-time RT-PCR, showed a 50% inhibitory R64 concentration between 0.588 and 0.493 *μ*M, respectively.

In summary, based on previous work [14, 15, 18, 26], we have designed a 64-residue peptide (R64, Figure 1(b)) containing a central HIV-1 enhancer recognition helix and terminal nuclear localization and plasma membrane transduction signals. This peptide bound to the two HIV-1 enhancers and thus competed with NF-*κ*B, the major activator of HIV-1 replication [46, 47]. After incubation of CHO-K1 cells with R64, the peptide could be localized to cytosol and nucleus of these cells. In CEM-GFP cells, it suppressed the HIV-1 enhancer-driven expression of the GFP gene after PMA stimulation and inhibited viral transcription and replication after HIV-1 infection in a concentration-dependent manner. Most likely, R64 displaced NF-*κ*B from its two binding sites in HIV-1 LTR despite the presence of NF-*κ*B binding sites on many cellular genes [48]. An artificial 62-residue peptide (R62, Figure 1(c)) which contained both membrane transduction signals at the N-terminus but otherwise was identical with R64, also bound to the HIV-1 LTR enhancer region. However, it was active only in a cell-free *in vitro* transcription assay and did not inhibit HIV-1 replication in infected cells.

**Figure 9:**
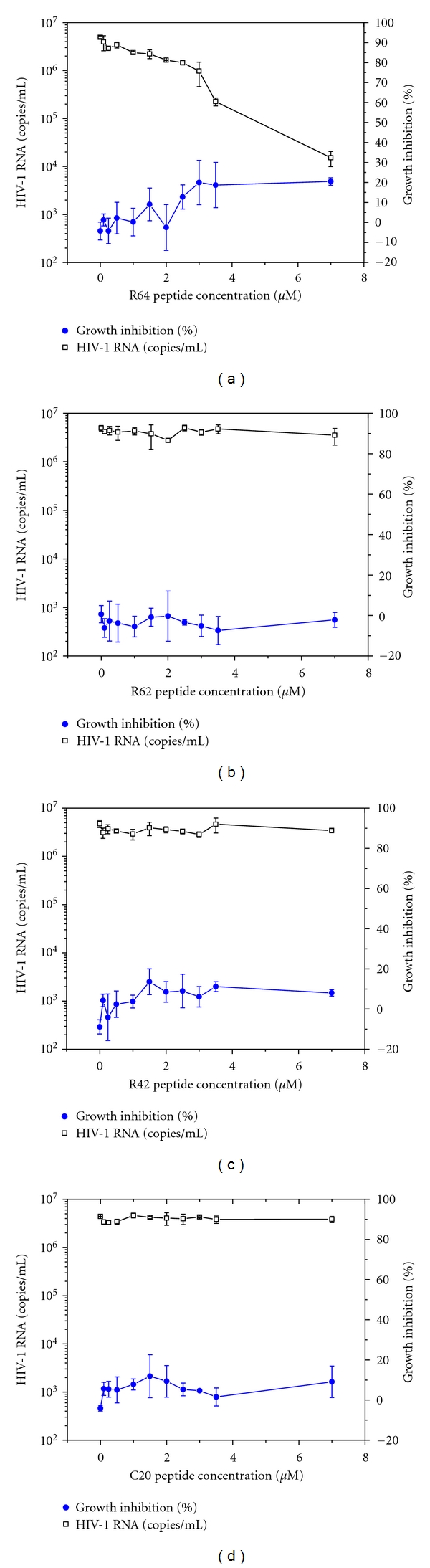
Assessment of HIV-1 RNA levels (□) and cell growth-inhibitory activity (●) in the culture supernatants of CEM-GFP cells at day eight after infection with HIV-1 and incubation with increasing concentrations of peptides R64 (a), R62 (b), R42 (c), and C20 (d). Peptides were added at day one after infection, their concentrations ranged from 0.1 *μ*M to 7 *μ*M. Growth inhibition was assessed using the MTT assay. The figure shows the results of two independent *in vitro* infection experiments with mean value ± standard deviation.

**Figure 10:**
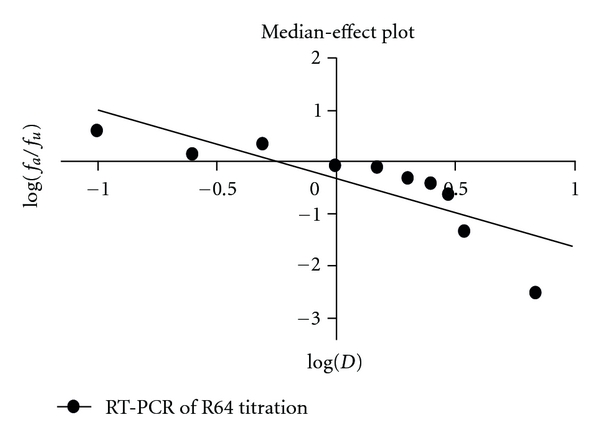
Determination of the 50% effective dose of peptide R64 based on R64 concentration-dependent HIV-1 RNA levels. Using the CalcuSyn 1.1 software package (Biosoft) [39], a median-effect plot was derived from the dose-effect curve obtained at day 8 after infection using the equation: *f*_*a*_/*f*_*u*_ = (*D*/*D*_*m*_)^*m*^ where *D* is the dose, *D*_*m*_ is the dose required for a 50% effect, *f*_*a*_ is the fraction affected by the dose, *f*_*u*_ is the unaffected fraction, *m* is a coefficient of the sigmoidicity of the dose-effect curve, and *r* is the correlation coefficient (*D*_*m*_ = 0.588 *μ*M, *r* = 0.8249, *m* = −1.320).

Therapeutic peptides have important applications in human diseases such as prevention of HIV-1 infection [2, 3], induction of tumor-specific cytotoxic T-lymphocytes [17], and as therapeutic vaccines [49]. R64 seemed to fulfil some of the requirements as a therapeutic peptide: the Western blots of the cytosolic and nuclear extracts of R64-incubated CHO-K1 cells (Figure 5) showed no fragments, indicating stability of the peptide against intracellular proteolytic degradation; R64 reduced the viral replication by almost three orders of magnitude and was not cytotoxic at a concentration of 7 *μ*M in the medium of HIV-1-infected CEM-GFP cells (Figure 9); and uptake of R64 into cells appeared to be fast (Figure 6), lowering the immunogenic potential of this peptide.

## Abbreviations

HIV-1: Human immunodeficiency virus type 1
LTR: Long terminal repeat
R42: Artificial 42-residue HIV-1 enhancer-binding peptide
NLS: Nuclear localization signal
PTD: Protein transduction domain
GFP: Green fluorescent protein
NF-*κ*B: Nuclear factor *κ*B
Sp1: Specificity protein 1
PMA: Phorbol-12-myristate-13-acetate
MTT: 3-[4,5-dimethylthiazol-2-yl]-2,5-diphenyltetrazolium bromide.
Bp: Base pair.
